# Inhibition of the angiopoietin/Tie2 axis induces immunogenic modulation, which sensitizes human tumor cells to immune attack

**DOI:** 10.1186/s40425-015-0096-7

**Published:** 2015-11-17

**Authors:** Italia Grenga, Anna R. Kwilas, Renee N. Donahue, Benedetto Farsaci, James W. Hodge

**Affiliations:** Laboratory of Tumor Immunology and Biology, Center for Cancer Research, National Cancer Institute, National Institutes of Health, Center Drive, Room 8B13 MSC 1750, Bethesda, MD 20892 USA

**Keywords:** Angiopoietin, Tie2, Immunogenic modulation, Immunotherapy

## Abstract

**Background:**

The angiopoietin/Tie2 pathway is an attractive target for cancer therapy due to its well-known role in regulating angiogenesis. Trebananib, a recombinant peptide-Fc fusion protein, or peptibody, that binds to angiopoietin-1 (Ang1) and Ang2 to block their interaction with the Tie2 receptor, is under active clinical investigation. We investigated whether suppressing the angiopoietin/Tie2 pathway, using the preclinical version of Trebananib (mL4-3 and L1-7(N)), could increase the sensitivity of human tumor cells to immune-mediated lysis through immunogenic modulation, which would make Trebananib a promising candidate for combination with immunotherapy.

**Methods:**

We assessed human carcinoma cells for expression and activation of Ang1 and Ang2 and their receptor tyrosine kinase Tie2. In vitro, we exposed tumor cell lines expressing Tie2 to the peptibodies mL4-3 and L1-7(N), which inhibit the binding of Ang1 and Ang2 to Tie2, and assessed the cells for changes in viability, proliferation, surface phenotype, and sensitivity to attack by antigen-specific cytotoxic T lymphocytes (CTLs).

**Results:**

Suppression of the angiopoietin/Tie2 pathway using mL4-3 and L1-7(N) had no effect on the proliferation or viability of tumor cells. However, these inhibitors markedly altered tumor cell phenotype, rendering tumor cells significantly more sensitive to antigen-specific CTL killing. ICAM-1 was shown to be mechanistically involved in these inhibitors’ ability to sensitize tumor cells to immune-mediated attack by functional blocking studies.

**Conclusion:**

Our findings provide a rationale for the combination of agents targeting the angiopoietin/Tie2 pathway with cancer immunotherapies.

## Background

While accumulating evidence demonstrates that cancer vaccines are safe, their clinical efficacy needs to be improved. To that end, recent studies have shown that certain antiangiogenic therapies have immunomodulatory effects, both peripherally and within the tumor microenvironment, that make them promising candidates for combination with cancer immunotherapy [1–6]. For example, the tyrosine kinase inhibitors (TKIs) sunitinib and sorafenib have been shown to alter the immune landscape, increasing the frequency and function of effector immune elements while decreasing the number or function of immune suppressor cells [7–9]. These agents also reduce solid tumor pressure by decreasing tumor compactness and tight junctions, allowing for improved perfusion of collapsed vessels and increased tumor oxygenation, both of which are vital to the function of immune cells [8]. Recently an additional TKI, cabozantinib, an inhibitor of multiple receptor tyrosine kinases, including RET, MET and VEGFR2 [10, 11], has been reported to induce immunogenic modulation, altering tumor-cell phenotype and sensitizing tumor cells to immune-mediated attack [3]. In preclinical models, the immunomodulatory actions of sunitinib, sorafenib, and cabozantinib have had synergistic antitumor effects when combined with immunotherapy [3].

Angiogenesis and vascular remodeling are complex processes that involve regulation by the cytokines angiopoietin-1 (Ang1) and Ang2, which function through their interaction with the vascular receptor tyrosine kinase Tie2 [12, 13]. Ang1 and Ang2 were originally described as have opposing effects, with Ang1 functioning as a Tie2 agonist contributing to vessel stabilization and maturation [14], and Ang2 functioning as an antagonist leading to vessel destabilization [15, 16]. However, studies have since shown that, like Ang1, Ang2 can induce Tie2 receptor phosphorylation and promote chemotaxis, tube formation, migration, and sprouting of endothelial cells in the absence of Ang1, demonstrating that Ang2 can also act as an agonist of Tie2 [13, 17, 18]. Ang2 is upregulated at sites of tumor angiogenesis. Additionally, overexpression of Ang2 accelerated tumor growth and angiogenesis in some experimental cancer models [19]. Clinically, high levels of Ang1 and/or Ang2 are reported in patients with breast, gastric, ovarian, mesothelioma, and non-small cell lung cancer and, in some cases, are associated with higher-stage disease and poor clinical outcome [20–26].

The central role of the angiopoietin/Tie2 signaling pathway in regulating angiogenesis makes it an attractive target for cancer therapies. Suppression of the Tie2 pathway using Trebananib, an investigational recombinant peptide-Fc fusion protein, or peptibody, that selectively binds to Ang1 and Ang2 and blocks their interaction with Tie2, is under active clinical investigation in various cancers, including breast, prostate, and ovarian. Trebananib has been modeled in preclinical studies by combining two peptibodies, mL4-3 and L1-7(N), which inhibit Ang1 and Ang2, respectively. In a xenograft model of colon cancer, systemic administration of mL4-3 and L1-7(N) provided greater antitumor activity than either agent alone, and was mechanistically involved in reducing and rearranging tumor vasculature [15, 27]. Although Tie2 was originally described as an endothelial cell-specific receptor [28], it has since been identified in monocytes and non-stromal cells in thyroid, breast, and gastric cancers [29–31]. Tie2 has also been identified in a human glioma cancer cell line and was autophosphorylated, suggesting constitutive activity of the Tie2 pathway in these cells [32].

The present study investigated whether inhibiting the Tie2 pathway in human breast, colon, prostate, and ovarian cancer cell lines could increase the sensitivity of tumor cells to immune-mediated lysis through immunogenic modulation. To our knowledge, this is the first study to report the novel immunomodulatory effects of targeting Tie2 directly on tumor cells. Inhibition of the angiopoietin/Tie2 pathway, using the peptibodies mL4-3 and L1-7(N) to neutralize the binding of Ang1 and Ang2 to Tie2, rendered human carcinoma cell lines of the breast, prostate, and ovary significantly more sensitive to T cell-mediated attack. These findings, in addition to the reported capacity of mL4-3 and L1-7(N) to modulate tumor angiogenesis and induce antitumor activity preclinically, provide a rationale for combining agents targeting the angiopoietin/Tie2 axis with cancer immunotherapy.

## Results

### Select human tumor cells express Tie2, Ang1, and Ang2 mRNA

**Fig. 1:**
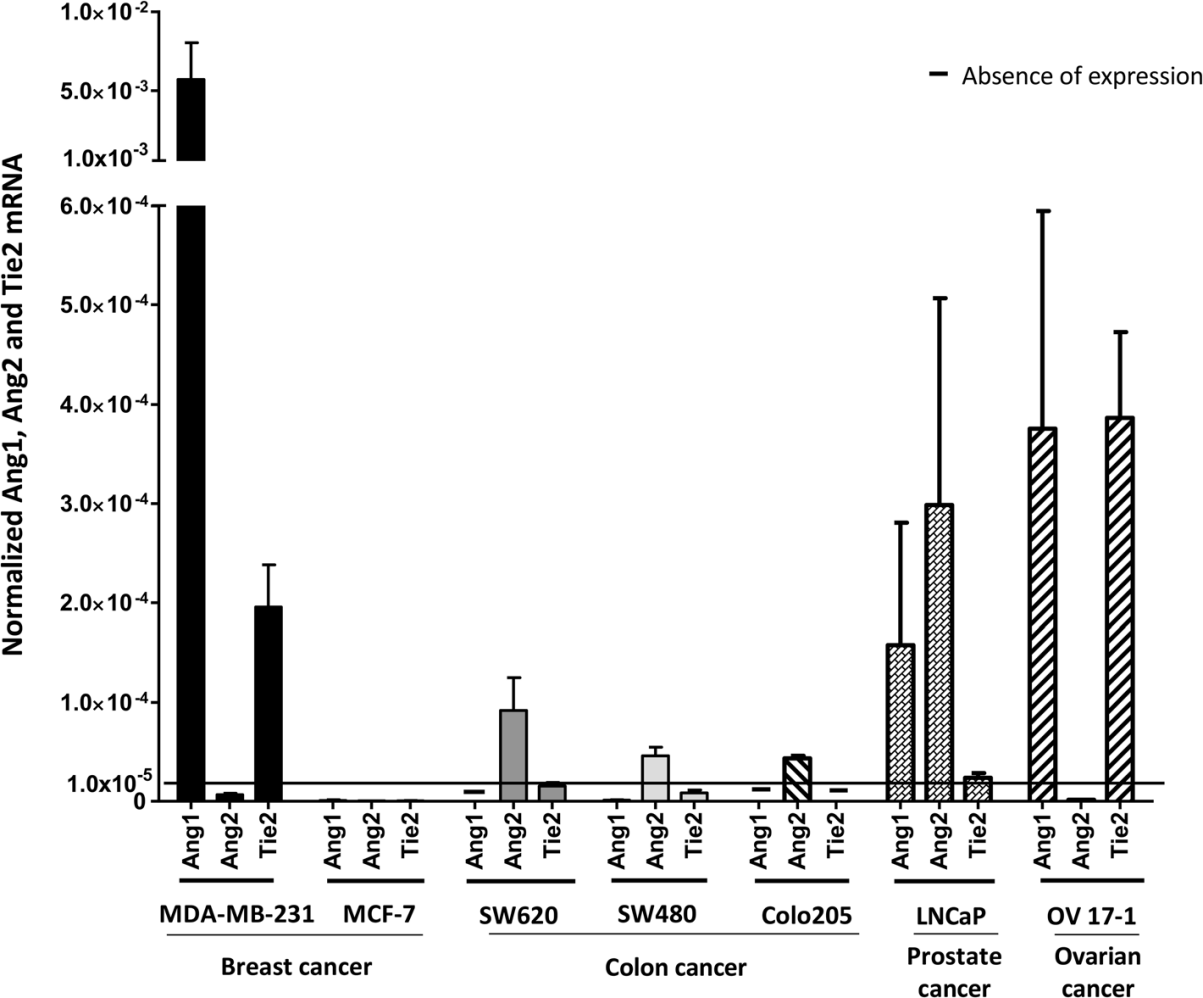
RNA expression of Ang1, Ang2, and Tie2 in human tumor cell lines. Seven human tumor cell lines were evaluated by RT-PCR for RNA expression of Ang1, Ang2, and Tie2. Data were normalized to GAPDH. Bars indicate mean ± SEM

To analyze expression of Tie2, Ang1, and Ang2, 2 breast (MDA-MB-231 and MCF-7), 3 colon (SW620, SW480, and Colo205), 1 prostate (LNCaP), and 1 ovarian (OV17-1) human tumor cell lines were tested by RT-PCR. Cells showed varying levels of Tie2, Ang1, and Ang2 mRNA. Among the cell lines analyzed, only MDA-MB-231, LNCaP, and OV17-1 had detectable levels of Tie2 (Fig. 1). These same cell lines also expressed Ang1; however, Ang2 was only expressed in LNCaP cells. Cell lines expressing Tie2 and either Ang1 or Ang2 were selected for further study.

### The Tie2 pathway is functional in breast and ovarian tumor cells

**Fig. 2:**
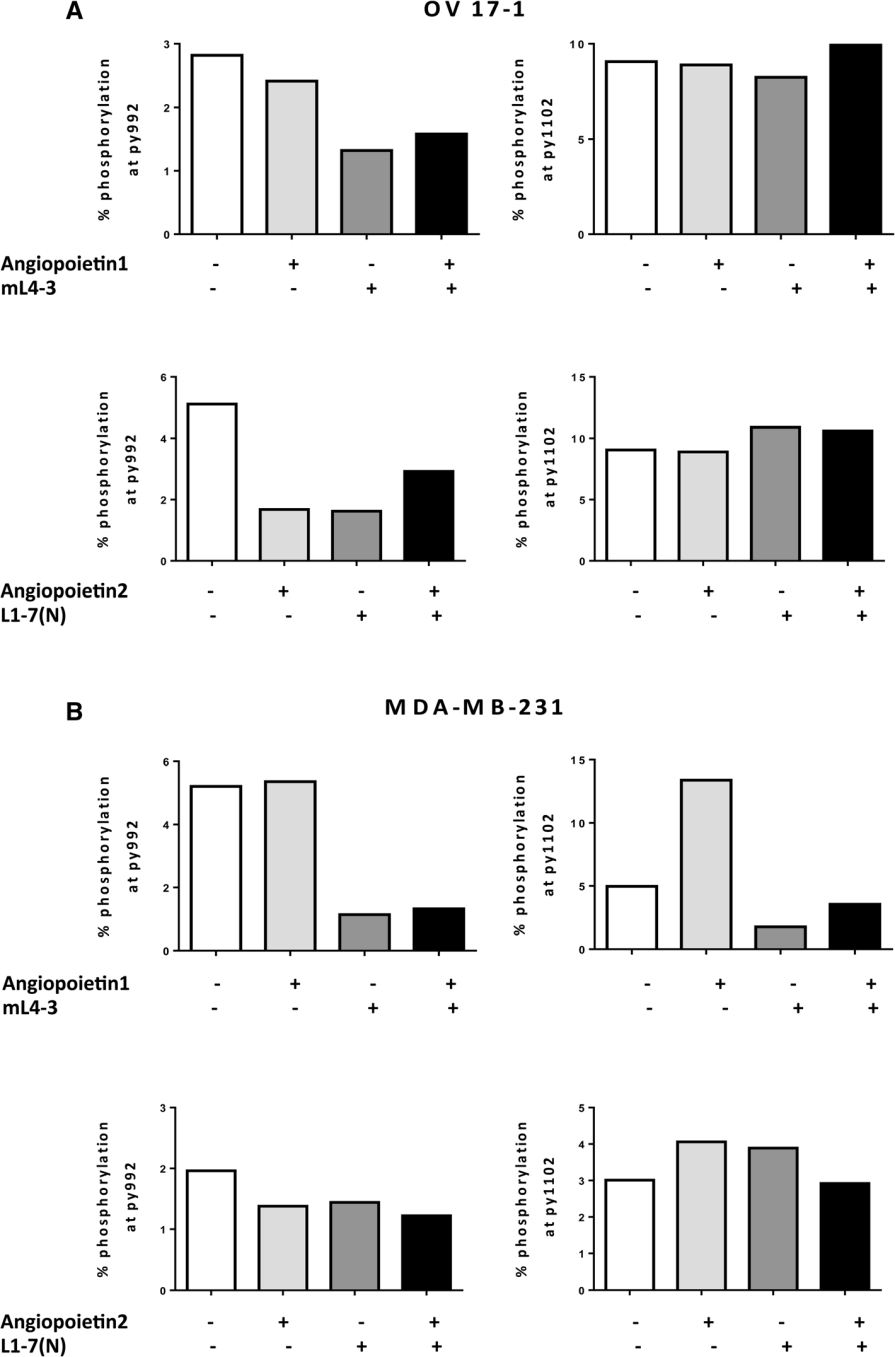
Activation of Tie2 pathway in human tumor cell lines. Tie2 phosphorylation at sites py992 and py1102 and the ability of Ang1 (mL4-3) and Ang2 (L1-7(N) inhibitors to reduce activation of the Tie2 pathway were evaluated by flow cytometry in OV17-1 (**a**) and MDA-MB-231 (**b**) tumor cell lines. A range of concentrations of mL4-3 (0.017–170 μg/mL) and L1-7(N) (0.0407–407 μg/mL) were tested, with no dose-dependent effects noted. Data are shown at 17 μg/mL and 4.07 μg/mL for mL-4-3 and L1-7(N), respectively. Exogenous recombinant Ang1 or Ang2 (2 μg/mL) was added where indicated. Tumor cells were treated for 30 min at 37 °C, then harvested and stained to assess Tie2 phosphorylation

To investigate the activity of the Tie2 pathway in human tumor-cell lines, we assessed OV17-1 and MDA-MB-231 for 2 sites of phosphorylation in Tie2—py992 and py1102—reported to be important in endotheliocytes [33]. We measured phosphorylation of Tie2 in cultures treated with the Ang1 inhibitor mL4-3, either alone or in combination with exogenous recombinant Ang1, and in cells treated with the Ang2 inhibitor L1-7(N), either alone or in combination with exogenous recombinant Ang2. In OV17-1 cells (Fig. 2a), phosphorylation at site py992 was reduced by the addition of mL4-3 or L1-7(N). Furthermore, this reduction in phosphorylation was maintained when recombinant Ang1 or Ang2 was added to the cultures in the presence of each inhibitor. No alterations in phosphorylation at site py1102 were noted in OV17-1 cultures. In MDA-MB-231 cells (Fig. 2b), mL4-3 reduced Tie2 phosphorylation at both the py992 and py1102 sites. This reduction was maintained when exogenous Ang1 was added to the cells in the presence of the inhibitor. L1-7(N) did not alter phosphorylation in MDA-MB-231 cells at either site analyzed. These results indicate that the Tie2 pathway is active in OV17-1 and MDA-MB-231 cultures, with Ang1 and Ang2 functioning through the py992 site in OV17-1 cells and Ang1 functioning through both py992 and py1102 phosphorylation sites in MDA-MB-231 cells.

### Ang1 and Ang2 inhibitors do not alter cell proliferation and viability in human tumor cells

**Fig. 3:**
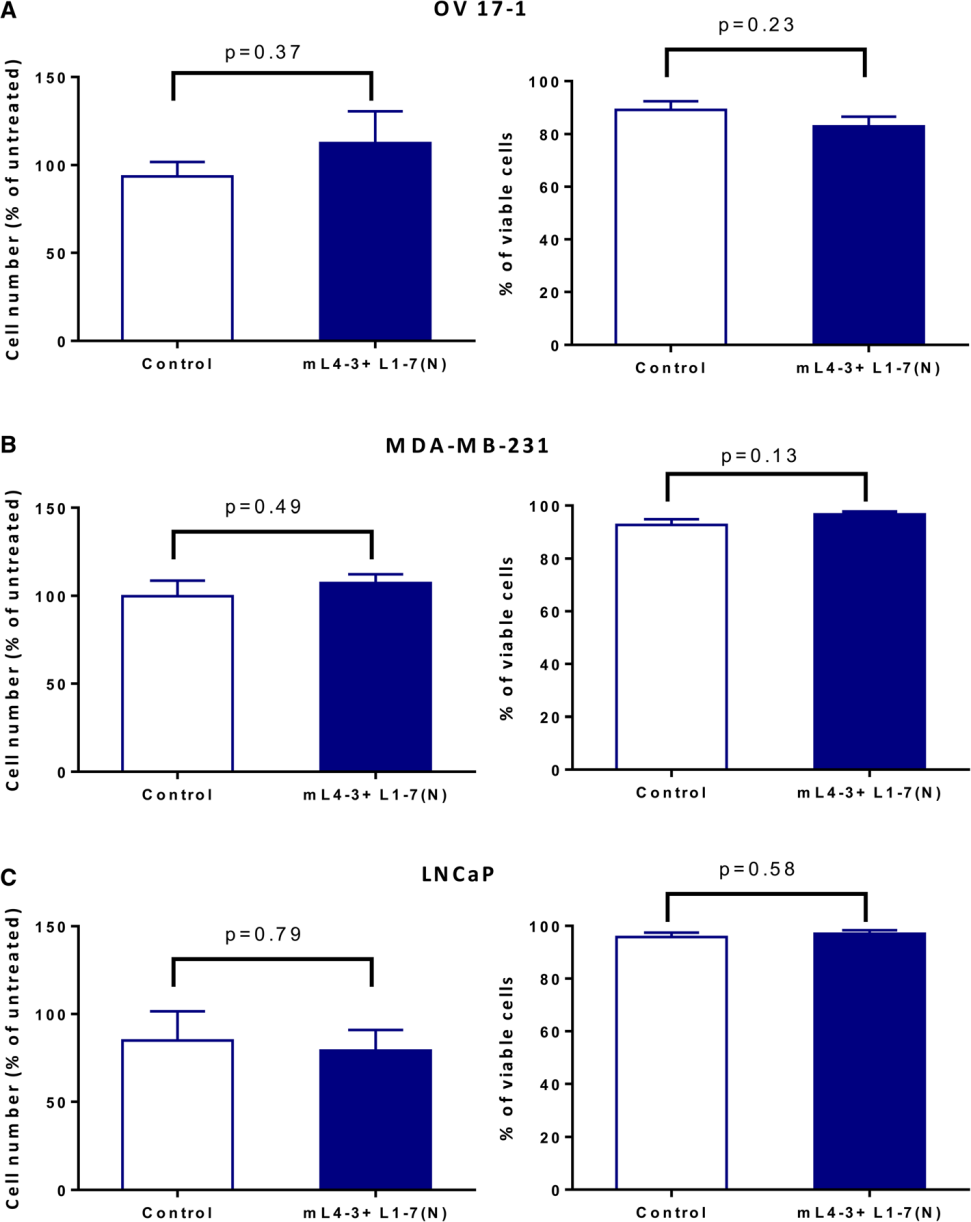
Ang1 and Ang2 inhibitors did not affect tumor-cell number or viability. Human ovarian cancer cells (OV17-1; **a**), breast cancer cells (MDA-MB-231; **b**) and prostate cancer cells (LNCaP; **c**) were treated with the Cmax of mL4-3 and L1-7(N) (16 and 10 μg/mL, respectively) or control (human IgG1-Fc, 26 μg/mL) for 3 days. Cells were then harvested, counted, and assessed for viability via trypan blue exclusion. Cell number is reported as a percentage of untreated cells. Data were analyzed with an unpaired *t* test. p values are indicated

To determine whether inhibition of the angiopoietin/Tie2 axis has a direct proliferative or cytotoxic effect on human tumor cells, OV17-1, MDA-MB-231, and LNCaP tumor cells were treated in vitro for 3 days with the Cmax of mL4-3 and L1-7(N) (16 and 10 μg/mL, respectively). Both untreated and Fc-treated cells (human IgG1-Fc at 26 μg/mL) served as controls. Relative to controls, cell number and viability were not significantly altered by treatment with mL4-3 and L1-7(N) in OV17-1, MDA-MB-231, and LNCaP cultures (Fig. 3).

### Ang1 and Ang2 inhibitors induce immunogenic modulation of human carcinoma cells

It has previously been shown that treatment with certain TKIs can modulate the phenotype of immunologically relevant molecules on tumor cells, making them more sensitive to T cell-mediated killing in a process known as immunogenic modulation [3]. To examine the potential of Ang1 and Ang2 inhibitors to alter tumor phenotype, OV17-1 and MDA-MB-231 cell cultures were exposed for 3 days to the Cmax of mL4-3 and L1-7(N) (16 and 10 μg/mL, respectively) or Fc control (human IgG1-Fc at 26 μg/mL) and then analyzed for expression of human leukocyte antigen (HLA)-A2, carcinoembryonic antigen (CEA), mucin (MUC)-1, ICAM-1 (CD54), calreticulin, Fas (CD95), Trail-R1, and Trail-R2. These molecules appear to enhance antitumor T-cell responses through various mechanisms [34–38]. Relative to controls, treatment with mL4-3 and L1-7(N) increased expression of ICAM-1, Fas, and Trail-R1 in both OV17-1 and MDA-MB-231 cell lines. CEA and Trail-R2 increased only in the OV17-1 cultures, while MUC-1 and calreticulin were upregulated only in the MDA-MB-231 cultures (Table 1). Among all the molecules examined, ICAM-1 was most robustly altered (42 % increase in mean fluorescence intensity (MFI)) following treatment in OV17-1 cultures, while calreticulin had the greatest increase in percentage (50 %) following treatment in MDA-MB-231 cells.

**Table 1:** Treatment with Ang1 and Ang2 inhibitors modulates the phenotype of human tumor cells

A. OV 17-1								
	HLA-A2	CEA	MUC-1	CD54	Calreticulin	CD95	Trail-R1	Trail-R2
	% (MFI)	% (MFI)	% (MFI)	% (MFI)	% (MFI)	% (MFI)	% (MFI)	% (MFI)
Control	99.4(34250)	40.7(731)	55.9(1170)	93.8(16581)	3.5(431)	57.2(691)	27.4(604)	10.1(93)
mL4-3 + L1-7(N)	99.1(34180)	40.0(**872**)	59.0(1124)	97.0(**23584**)	3.7(429)	**65.3(813)**	**33.7(750)**	10.1(**107**)
B. MDA-MB-231								
	HLA-A2	CEA	MUC-1	CD54	Calreticulin	CD95	Trail-R1	Trail-R2
	% (MFI)	% (MFI)	%(MFI)	% (MFI)	% (MFI)	% (MFI)	% (MFI)	% (MFI)
Control	98.7(62083)	40.2(671)	56.0(2268)	97.9(30985)	10.6(377)	35.1(438)	44.0(775)	35.1(367)
mL4-3 + L1-7(N)	99.1(60495)	43.7(666)	59.4(**2670**)	99.1(**35652**)	**15.9(428)**	**41.2**(493)	**48.7**(797)	30.5(292)

The human ovarian cancer cell line OV17-1 (A), and human breast cancer cell line MDA-MB-231 (B) were treated with the Cmax of mL4-3 and L1-7(N) (16 and 10 μg/mL, respectively) or control (human IgG1-Fc at 26 μg/mL) for 3 days

Cells were then harvested and analyzed by flow cytometry for expression of surface markers reported to be involved in CTL lysis (HLA-A2, CEA, MUC-1, ICAM-1, calreticulin, Fas, Trail-R1 and Trail-R2). Data indicate percentage of positive cells; MFI is in parentheses. Gating was performed using isotype controls Bold values indicate marker upregulation of > 10 % in percentage or MFI compared to controls

### Ang1 and Ang2 inhibitors increase the sensitivity of human tumor cell lines to T cell-mediated killing

**Fig. 4:**
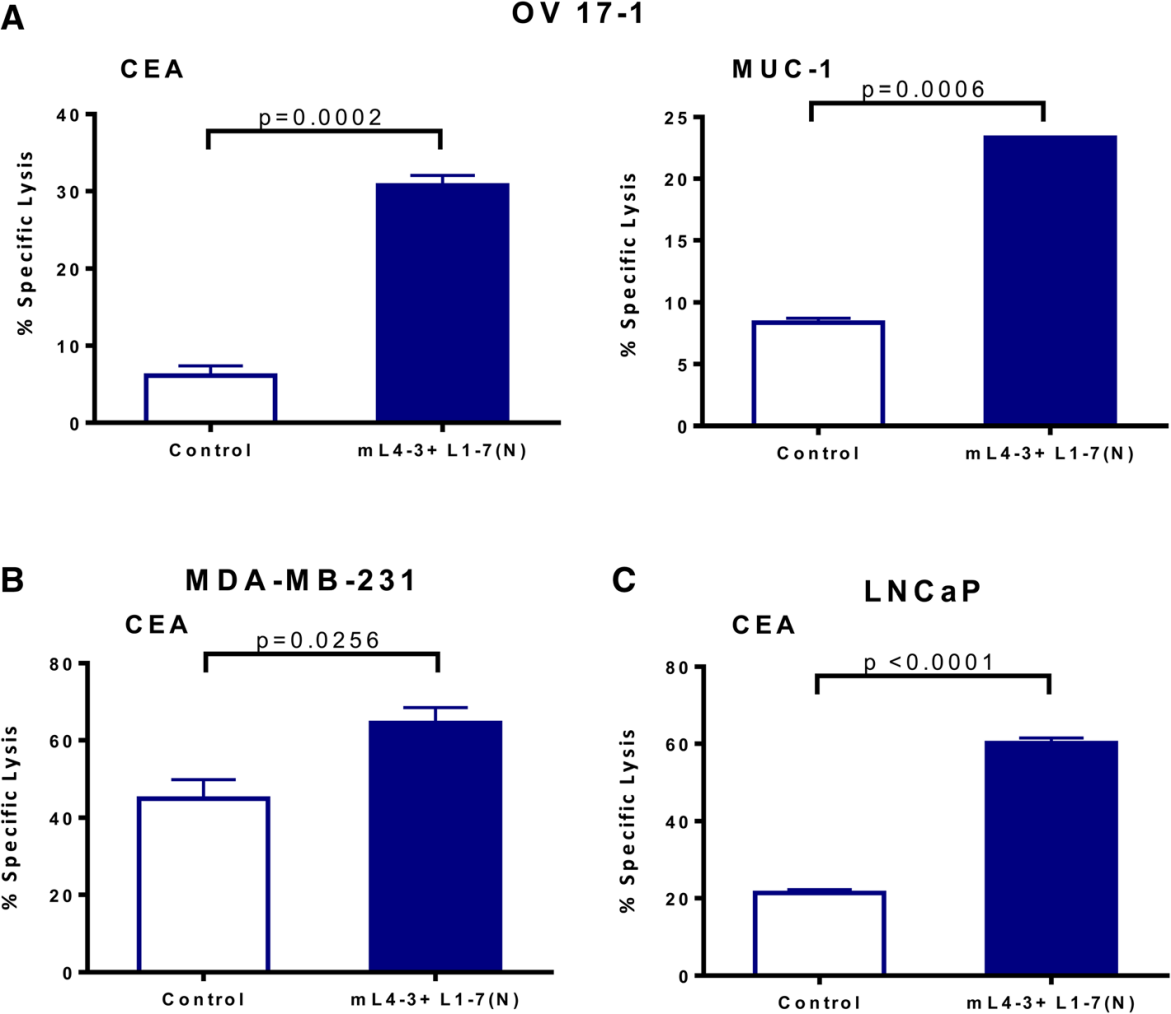
Ang1 and Ang2 inhibitors increased CTL-mediated lysis of human tumor cells. Human ovarian cancer cells (OV17-1; **a**), breast cancer cells (MDA-MB-231; **b**) and prostate cancer cells (LNCaP; **c**) were treated with the Cmax of mL4-3 and L1-7(N) (16 and 10 μg/mL, respectively) or control (human IgG1-Fc at 26 μg/mL or no treatment) for 3 days. Cells were labeled with ^111^In and co-incubated with CEA-specific CD8^+^ T cells at an effector: target ratio of 30:1 or MUC-1-specific CD8^+^ T cells at an effector:target ratio of 15:1 for 18 h. Effectors are indicated in the graphs. Bars indicate mean ± SEM. Statistical analyses were performed with an unpaired *t* test. *p* values < 0.05 were considered significant

To determine the functional significance of the phenotypic changes induced by Ang1 and Ang2 inhibitors, we next evaluated the potential of mL4-3 and L1-7(N) to modify the sensitivity of human tumor cells to lysis by CD8^+^ cytotoxic T lymphocytes (CTLs). OV17-1, MDA-MB-231, and LNCaP cells were exposed for 3 days to mL4-3 and L1-7(N) and then used as targets in a CTL killing assay. OV17-1 cells that were untreated or treated with the Fc control were killed by CEA- and MUC-1-specific T cells at a low level (Fig. 4a). Pretreatment of these targets with the Ang1 and Ang2 inhibitors increased killing by CEA- and MUC-1-specific T cells 5.1- and 2.8-fold, respectively. MDA-MB-231 and LNCaP cultures that were untreated or treated with the Fc control were lysed by CEA-specific CTLs at a level of 45 and 21 %, respectively. However, upon treatment with mL4-3 and L1-7(N), MDA-MB-231 and LNCaP targets were killed to a greater extent by CEA-specific T cells, with levels of 65 and 60 % lysis, respectively. These data indicate that exposing a variety of human tumor cells to Ang1 and Ang2 inhibitors enhances antigen-specific CTL-mediated killing, and that this effect extends to more than one tumor-associated antigen (TAA).

### ICAM-1 is part of the mechanism by which Ang1 and Ang2 inhibitors mediate immunogenic modulation in OV17-1 cells

**Fig. 5:**
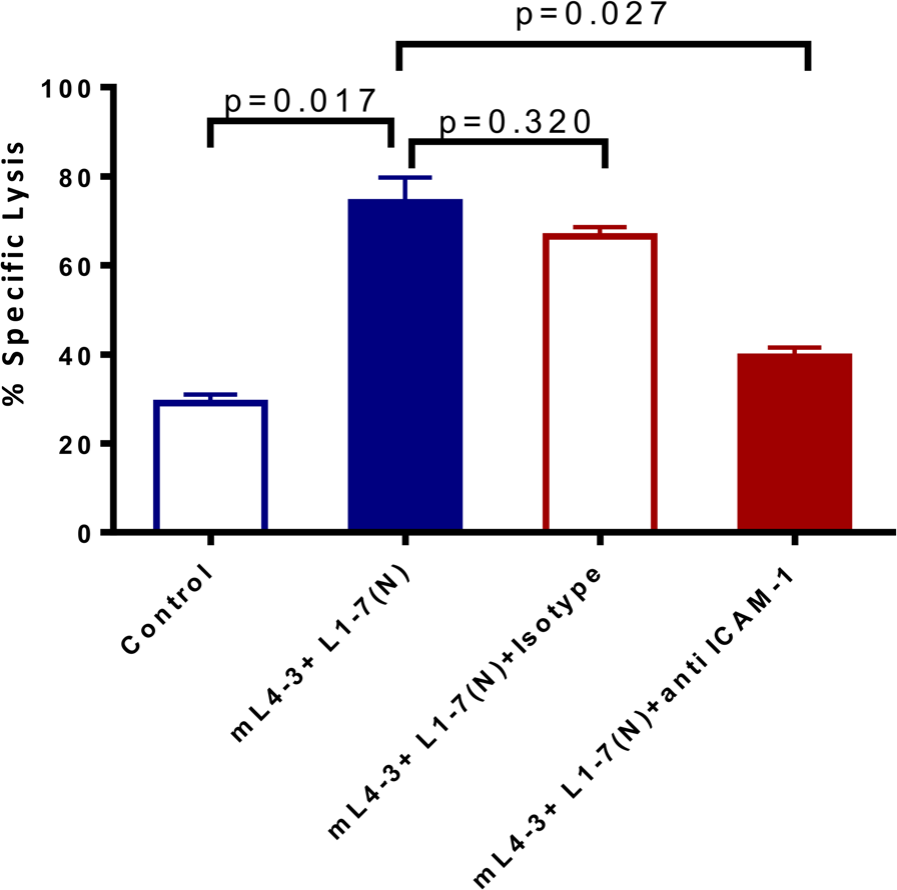
ICAM-1 blockade reduced the enhanced CTL lysis noted with Ang1 and Ang2 inhibitors. Human ovarian cancer cells (OV17-1) were treated with the Cmax of mL4-3 and L1-7(N) (16 and 10 μg/mL, respectively) or control (no treatment) for 3 days. Tumor cells treated for 3 days with Ang1 and Ang2 inhibitor were then pretreated for 1 h and co-incubated during the CTL assay with anti-ICAM-1 antibody (10 μg/mL) or the corresponding isotype control. Cells were labeled with ^111^In and co-incubated with CEA-specific CD8^+^ T cells at an effector: target ratio of 30:1 for 18 h. Bars indicate mean ± SEM. Statistical analyses were performed with an unpaired *t* test. p values < 0.05 were considered significant

To investigate an association between the increased ICAM-1 expression and improved CTL lysis observed following treatment with Ang1 and Ang2 inhibitors in OV17-1 cultures, we included a blocking antibody against ICAM-1 in a CTL killing assay. Pretreating tumor-cell targets with mL4-3 and L1-7(N) significantly increased lysis of OV17-1 cultures by CEA-specific T cells (from 29 to 74 %), while the addition of an anti-ICAM-1 blocking antibody, but not the corresponding isotype control, significantly reduced lysis (from 74 to 40 %) (Fig. 5). These data demonstrate that ICAM-1 is involved in the mechanism by which treatment with Ang1 and Ang2 inhibitors increase CTL-mediated lysis of tumor cells. However, while blockade of ICAM-1 in Ang1 and Ang2 treated cultures reduced lysis, it did not completely abrogate T cell mediated killing to pre-treatment levels, with a trend of a difference (*p* = 0.051) noted between these groups, suggesting that other potential mechanisms of action may be involved.

## Discussion

The central role of the angiopoietin/Tie2 signaling pathway in regulating angiogenesis makes it a therapeutically attractive target for cancer therapies. The relatively recent finding that Tie2 can be expressed in non-endothelial cells, including tumor cells [28], has led to renewed interest in the role of the angiopoietin/Tie2 pathway in cancer progression. The present study investigated whether suppressing the angiopoietin/Tie2 pathway in tumor cells could increase the sensitivity of tumor cells to immune-mediated lysis through immunogenic modulation. In this study, we first determined the expression of Ang1 and 2, as well Tie2, in a panel of human tumor cell lines, and then examined the functional consequence of inhibiting the angiopoietin/Tie2 pathway only in cells that expressed Tie2 and either Ang1 or 2. We show for the first time that human breast (MDA-MB-231), prostate (LNCaP), and ovarian (OV17-1) cancer cell lines express measureable levels of Tie2 and varying levels of Ang1 and Ang2 (Fig. 1). In addition, two of the cell lines analyzed showed activation of the Tie2 pathway (Fig. 2), with phosphorylation detected at sites of Tie2 that have been previously described in endothelial cells [13, 33, 39]. Specifically, in OV17-1 cultures both Ang1 and Ang2 signaled through Tie2, and inhibitors of Ang1 (mL4-3) or Ang2 (L1-7(N)) reduced phosphorylation at site py992, but not py1102, of Tie2. In contrast, in MDA-MB-231 cells only Ang1 signaled through Tie2, and mL4-3 reduced phosphorylation at sites py992 and py1102 of Tie2.

To date, the Ang1 and Ang2 inhibitors mL4-3 and L1-7(N) have been tested primarily in vivo. They have been shown to suppress tumor growth in mouse xenograft models and to modulate angiogenesis by reducing tumor vessels and normalizing remaining vessels [27, 40]. Here we demonstrate that treatment of human tumor cell lines in vitro with inhibitors of Ang1 and Ang2 does not alter cell number or viability (Fig. 3), indicating that these agents are not directly cytotoxic. However, treatment of cells with the combination of mL4-3 and L1-7(N) modulated tumor-cell phenotype, increasing the surface expression (either percentage or MFI) of CEA, MUC-1, ICAM-1, Fas, Trail-R1, Trail-R2, and calreticulin (Table 1). Each of these immunologically relevant molecules is involved in the cell-to-cell interaction by which CTLs kill tumor cells [35–38]. For example, ICAM-1 is a surface glycoprotein with both costimulatory and adhesive properties. Increased ICAM-1 levels have been correlated with CTLs binding to tumor cells and enhancing cytotoxicity [41–43]. Calreticulin is an intracellular protein that translocates to the cell surface in response to several stimuli, acting as a phagocytic signal for dendritic cells and making antigen presentation more efficient. Translocation of calreticulin to the outer membrane of dying cells was originally described to occur during immunologic cell death [44, 45]; however, translocation of this molecule to the cell surface has also been described to occur in non-dying cells that are undergoing immunogenic modulation [36], where exposure of tumor cells to certain chemotherapies and radiation, induces phenotypic changes in viable cells that makes them more susceptible to immune mediated killing. Upregulation of TAAs such as CEA and MUC-1 in tumor cells may also increase the efficiency of immune surveillance.

Consistent with the observed phenotypic modification of tumor cells by Ang1 and Ang2 inhibitors, we show for the first time that the sensitivity of tumor cells to antigen-specific T-cell lysis significantly increased in vitro (Fig. 4). Furthermore, in human ovarian cancer cells (OV17-1) a neutralizing antibody blocking ICAM-1 ameliorated the enhanced CTL lysis seen with Ang1 and Ang2 inhibitors, demonstrating that ICAM-1 was mechanistically involved in the increase in antigen-specific T-cell lysis (Fig. 5). It is likely, however, that upregulation of ICAM-1 is not the only mechanism by which blockade of Ang1 and Ang2 increased the sensitivity of tumor cells to CTL mediated lysis. Blockade of ICAM-1 in cells treated with Ang1 and Ang2 did not completely abrogate T cell mediated killing to pre-treatment levels, leaving room for other mechanisms. Additionally, as the adhesive interaction of ICAM-1 with LFA-1 plays a key role in formation of the immunologic synapse, blockade of ICAM-1 is well known to prevent formation of the immunologic synapse, and thus the binding of death ligands to death receptors. We show for the first time that inhibition of the angiopoietin/Tie2 axis in human tumor cells, using the preclinical version of Trebananib, induces immunogenic modulation and increases the susceptibility of tumor cells to antigen-specific lysis. Determining whether exposure of human tumor cells to Ang1 and Ang2 inhibitors similarly increases their susceptibility to non-antigen-specific and/or non-immune-mediated killing will require further testing. Additionally, as the present study examined the functional repercussion of inhibiting the angiopoietin/Tie2 axis only in cell lines that expressed this pathway, the present study does not preclude the possibility that Trebananib could have off target effects that can be exploited by T cells.

A number of antiangiogenic therapies that target VEGF and or its receptors, including bevacizumab, sorafenib, sunitinib, and cabozantinib, have shown clinical benefit as monotherapies in several different cancers. Bevacizumab, a recombinant humanized monoclonal antibody that blocks angiogenesis by inhibiting VEGF-A, has been approved by the U.S. Food and Drug Administration as a treatment for ovarian cancer [46, 47]. Like the VEGF pathway, the angiopoietin pathway involves a tyrosine kinase receptor, Tie2, which was initially thought to be expressed only in vascular endothelial cells [48]. Trebananib, the most clinically advanced inhibitor of both Ang1 and Ang2, is currently being tested in three phase III studies in advanced ovarian cancer (TRINOVA-1, NCT01204749; TRINOVA-2, NCT01281254; TRINOVA-3, NCT01493505), as well as in phase II studies of breast (NCT00511459, NCT01042379) and prostate cancer (NCT01553188).

The initial rationale for using antiangiogenic agents to treat cancer was to starve the tumor of oxygen and nutrients, thereby inhibiting its ability to grow and spread [49]. However, it has become increasingly clear that hypoxia actually promotes tumor invasiveness and metastasis [50]. Hence, it is increasingly thought that antiangiogenic therapies may actually normalize the abnormal structure and function of tumor vasculature, increasing tumor oxygenation and preventing the unfavorable switch to a more metastatic phenotype [51]. Improved oxygenation within tumors is important for potential combination studies with immunotherapy, as it is well known that a hypoxic microenvironment polarizes inflammatory immune cells toward a suppressive phenotype and function. Thus, antiangiogenic treatments that normalize tumor vasculature may be an effective way to potentiate immunotherapies, which require activation of tumor antigen-specific T cells, T-cell access into the tumor, and an immune-supportive environment to sustain T-cell function [52]. Combining antiangiogenic agents with cancer vaccines or immune checkpoint inhibitors may be an effective strategy for increasing the efficacy of each individual agent.

In fact, recent studies have demonstrated the ability of a number of antiangiogenic agents, including sunitinib, sorafenib, and cabozantanib, to synergize with cancer vaccines to produce enhanced antitumor activity [53]. In addition to their well-known vascular remodeling properties, these agents were shown to mediate antitumor immune responses by altering the frequency or function of immune-cell subsets in the periphery or the tumor microenvironment [1, 3, 8]. Only one of these agents, cabozantinib, which is an inhibitor of multiple receptor tyrosine kinases, including RET, MET, and VEGRF2 has been reported to induce immunogenic modulation, altering tumor-cell phenotype and sensitizing tumor cells to immune-mediated attack [3]. To our knowledge, there are no data available on the direct effect of VEGF inhibition, e.g., with an agent such as bevacizumab, on the sensitivity of human tumor cells to immune mediated lysis. The results of this present study, demonstrating that inhibition of the angiopoietin/Tie2 axis with the preclinical version of Trebananib (mL4-3 and L1-7 (N)) upregulates immunologically relevant molecules and increases antigen-specific lysis of tumor cells, support the combination of Ang1 and Ang2 inhibitors such as Trebananib with cancer immunotherapy.

## Conclusion

This study revealed a novel antitumor effect of suppression of the angiopoietin/Tie2 axis. We show for the first time that human breast, prostate, and ovarian cancer cell lines express measureable levels of Tie2, and that treatment of these cells with the preclinical versions of Trebananib (mL4-3 and L1-7 (N)), which are selective peptibodies that inhibit the binding of Ang1 and Ang2 to Tie2, mediates immunogenic modulation, rendering tumor cells more susceptible to T cell-mediated killing. Thus, taking together Trebananib’s novel immune regulatory properties and well-known vascular remodeling properties, the present study supports the combination of Ang1 and Ang2 inhibitors with cancer immunotherapy to potentiate antitumor activity.

## Methods

### Tumor-cell lines

Human breast (MDA-MB-231 and MCF-7), colon (SW620, SW480, and Colo205), and prostate (LNCaP) tumor cell lines were purchased from American Type Culture Collection (Manassas, VA) and cultured according to the indicated protocol. The ovarian cancer cell line OV17-1, derived from primary ovarian tumor with endometrioid adenocarcinoma morphology, was obtained from Onyvax (London, UK). OV17-1 cells were cultured in complete RPMI medium (10 % fetal bovine serum, 100 units/mL penicillin, 100 μg/mL streptomycin, 2 mM L-glutamine). All cells were cultured in a humidified atmosphere at 37 °C with 5 % CO_2_.

### Drug preparation and treatment

XThe peptibodies mL4-3 and L1-7(N) were provided by Amgen (Thousand Oaks, CA) and stored at −80 °C. These agents were designed for preclinical studies to mimic the effect of Ttrebananib, the Ang1 and Ang2 inhibitor currently used in clinical trials. The drugs were diluted with medium immediately before use at the indicated concentrations. The Cmax of mL4-3 and L1-7(N) were calculated based on murine experiments [27]. Human recombinant IgG1-Fc protein (Life Technologies, Frederick, MD or ACROBiosystems, Newark, DE) or no treatment served as a control in all experiments.

### RT-PCR

Total RNA was isolated from breast (MDA-MB-231 and MCF-7), colon (SW620, SW480, and Colo205), prostate (LNCaP), and ovarian (OV17-1) tumor cell lines using the RNeasy extraction kit (Quiagen, Valencia, CA) and reverse-transcribed into cDNA with the Advantage RT-PCR kit (Clontech, Mountain View, CA). cDNA (100 ng) was evaluated by RT-PCR using primers for Ang1 (Hs00375822_m1), Ang2 (Hs01048042_m1), Tie2 (Hs00945146_m1), and the endogenous control glyceraldehyde 3-phosphate dehydrogenase (GAPDH) (4326317E) (Applied Biosystems, Carlsbad, CA). Expression of each gene of interest was normalized to GAPDH. The assay was performed using the 7300 RT-4CR System (Applied Biosystems).

### Tie2 phosphorylation assay

Ovarian (OV17-1) and breast (MDA-MB-231) tumor cells were plated in 6-well plates (2 × 10^5^ cells/well) and cultured with the recommended medium for 24 h. Tumor cells were then serum-starved for 24 h and subsequently treated for 30 min at 37 °C with logarithmic concentrations of the Ang1 inhibitor (mL4-3, 0.017–170 ng/mL) or Ang2 inhibitor (L1-7(N), 0.0407–407 ng/mL) with or without human recombinant Ang1 or Ang2 (2 μg/mL) (R&D Systems, Minneapolis, MN). Cells were then harvested and stained for Tie2 (AF700) (R&D Systems), py992 (AF647), and py1102 (PE) (BD Biosciences, San Diego, CA), following the manufacturer’s protocol for assessing phosphorylated intracellular proteins. Stained cells were acquired with an LSRII flow cytometer (BD Biosciences) and analyzed using FlowJo software (TreeStar, Inc., Ashland, OR).

### Assessment of cell proliferation and viability

To investigate the effect of Ang1 and Ang2 inhibitors on tumor-cell proliferation and viability, ovarian (OV17-1), breast (MDA-MB-231), and prostate (LNCaP) tumor cells were treated with the Cmax of mL4-3 and L1-7(N) (16 and 10 μg/mL, respectively) or control (human IgG1-Fc at 26 μg/mL) for 3 days. Cells were then harvested and counted via trypan blue exclusion.

### Assessment of phenotypic modulation

Ovarian (OV17-1) and breast (MDA-MB-231) tumor cells were treated with the Cmax of mL4-3 and L1-7(N) or control (human IgG1-Fc) for 3 days. Cells were then harvested and analyzed for changes in expression of a number of cell-surface markers that have been reported to be immunologically relevant [34–38]. Cells were stained for 30 min at 4 °C using the following antibodies: anti-CD54/ICAM-1-PE, anti-CD95/Fas-FITC, anti-CD227/MUC-1-FITC, anti-HLA-A2-PerCP-Cy5.5 (BD Biosciences), anti-Trail-R1-PerCP, anti-Trail-R2-AF700, anti-calreticulin-PE (R&D Systems), and anti-CEA-APC (Miltenyi Biotec, San Diego, CA); appropriate isotype controls were used. Live/dead-Pacific Blue (Life Technologies) was included to discriminate viable cells. Stained cells were acquired with an LSRII flow cytometer (BD Biosciences) and analyzed using FlowJo software (TreeStar, Inc.). Isotype staining was < 5 % for all samples analyzed. Proteins were defined as upregulated by treatment with the angiopoietin inhibitors if either the percentage of cells or the MFI increased by > 10 % relative to cells treated with the IgG1-Fc control.

### CTL assay

To evaluate the ability of Ang1 and Ang2 inhibitors to modify the sensitivity of tumor cells to lysis by CD8^+^ CTLs, ovarian (OV17-1), breast (MDA-MB-231), and prostate (LNCaP) tumor cells were treated with the Cmax of mL4-3 and L1-7(N) or control (human IgG1-Fc or no treatment) for 3 days. Tumor cells were labeled with ^111^In-labeled oxyquinoline and co-incubated with CEA-specific CD8^+^ T cells at an effector:target ratio of 30:1 or MUC-1-specific CD8^+^ T cells at an effector:target ratio of 15:1 for 18 h. CTLs were generated and utilized as described: CEA-specific CD8^+^ T cells recognize the CEA peptide epitope YLSGANLNL (CAP-1) [54], and MUC-1-specific CD8^+^ T cells recognize the MUC-1 peptide epitope ALWGQDVTSV [55]. These human CTL lines have been in culture for a long period of time, and as such their cytotoxic activity can be variable; however, each experiment was independently controlled. Following incubation of CTLs with tumor targets, supernatants were harvested and ^111^In release measured using a WIZARD 2 Automatic Gamma Counter (PerkinElmer, Waltham, MA). The percentage of tumor lysis was calculated using the formula: % tumor lysis = [(experimental cpm-spontaneous cpm)/(maximum cpm-spontaneous cpm)] x 100.

### CTL assay with ICAM-1 blocking antibody

Ovarian tumor cells (OV17-1) were treated with the Cmax of mL4-3 and L1-7(N) or control (no treatment) for 3 days. Cells were then harvested and pretreated for 1 h and co-incubated with an anti-ICAM-1 antibody (10 μg/mL) (BD Biosciences) or the corresponding isotype control during the CTL assay. The CTL killing assay was analyzed as previously described.

### Statistical analysis

Statistical analysis was performed using GraphPad Prism (GraphPad Software, La Jolla, CA). Differences between treatments were assessed using an unpaired Student’s *t* test with a 2-tailed distribution. Results are reported as p values calculated using a confidence interval of 95 % (*p* values < 0.05 are considered statistically significant).

## Abbreviations

Ang: Angiopoietin
TKI: Tyrosine kinase inhibitor
HLA: Human leukocyte antigen
CEA: Carcinoembryonic antigen
MUC: Mucin
MFI: Mean fluorescence intensity
CTL: Cytotoxic T lymphocyte
h: Hour
min: Minute
RT-PCR: Real-time polymerase chain reaction
VEGF: Vascular endothelial growth factor
GAPDH: Glyceraldehyde 3-phosphate dehydrogenase
